# National Trends in Sales and Price for Commercial Tobacco and Nicotine Products, 2018-2022

**DOI:** 10.1001/jamanetworkopen.2024.1384

**Published:** 2024-03-07

**Authors:** Ollie Ganz, Melanie LaVake, Mary Hrywna, Jessica L. King Jensen, Cristine D. Delnevo

**Affiliations:** 1 Rutgers Institute for Nicotine and Tobacco Studies, New Brunswick, New Jersey; 2 Department of Health Behavior, Society and Policy, Rutgers School of Public Health, Piscataway, New Jersey; 3 Department of Family Medicine and Community Health, Rutgers Robert Wood Johnson Medical School, New Brunswick, New Jersey

## Abstract

This cross-sectional study explores recent trends in sales and price of tobacco and nicotine products in the US.

## Introduction

Tobacco product pricing is an important marketing strategy.^1,2^ Tobacco price–sensitive populations include youth and young adults,^1^ with price-reducing promotions attributed to greater use.^1^ The current tobacco marketplace contains products across the continuum of harm, from combustible (ie, cigarettes and cigars) to noncombustible (ie, smokeless tobacco [SLT]) products, electronic nicotine delivery systems [ENDS]), and, more recently, modern oral nicotine (MON; eg, nicotine pouches). This cross-sectional study explores recent trends in sales and price of these products.

## Methods

Quarterly US tobacco sales data were licensed for convenience stores from the Nielsen Company (January 1, 2018, to December 31, 2022). Nielsen reports MON products as SLT; using brand, we disaggregated MON. For each product, we reported units sold and mean sale price (including taxes and discounts), adjusted for inflation (2022 US dollars). We calculated market share by product based on total units sold. Trends were assessed using Joinpoint, version 4.9.0 (National Cancer Institute), a segmented regression application, for average quarterly percentage changes (AQPCs). We present the best model fit, with each segment described by its short-term trend (quarterly percentage changes). Percentage changes with 95% CIs that did not cross 0 were considered significant (2-sided *P* < .05). Institutional review board approval and informed consent were exempted by Rutgers University because this study was not considered human participant research. We followed STROBE reporting guidelines.

## Results

From 2018 to 2022, cigarettes held the greatest market share (69.0%-60.4%), followed by cigars (19.1%-22.8%), SLT (10.3%-9.9%), ENDS (1.5%-3.4%), and MON (0.1%-3.4%) (Table). Total tobacco unit sales significantly decreased (AQPC, −0.4%; 95% CI, −0.7% to −0.1%), cigarette sales significantly decreased (AQPC, −1.1%; 95% CI, −1.5% to −0.8%), and ENDS (AQPC, 4.5%; 95% CI, 2.1- 7.0%) and MON (AQPC, 23.2%; 95% CI, 21.3%- 25.1%) sales significantly increased (Figure, A). ENDS sales increased rapidly during 2018 and then slowed; MON sales increased from 2018 to 2019, then slowed, but then continued to significantly increase.

**Table.** Tobacco Sales, Market Share, and Mean Price by Product Type in US Convenience Stores, 2018-2022^a^

	2018				2019				2020				2021				2022				2018-2022 AQPC (95% CI)
	Q1	Q2	Q3	Q4	Q1	Q2	Q3	Q4	Q1	Q2	Q3	Q4	Q1	Q2	Q3	Q4	Q1	Q2	Q3	Q4	
Total sales, units^b^	2.602	2.779	2.772	2.670	2.548	2.682	2.697	2.608	2.580	2.797	2.825	2.659	2.605	2.708	2.631	2.534	2.438	2.576	2.571	2.390	−0.4 (−0.7 to −0.1)^c^
Market share, %																					
Cigarettes	69.0	69.1	68.9	67.8	67.5	67.8	67.8	66.2	64.9	64.5	65.4	64.4	62.9	63.8	63.9	62.4	61.1	61.8	61.9	60.4	−1.1 (−1.5 to −0.8)^c^
Cigars	19.1	19.0	19.1	19.4	19.6	19.1	18.8	19.8	20.8	21.9	20.6	20.9	21.6	21.2	20.8	21.8	22.4	22.0	22.0	22.8	0.3 (−0.8 to 1.4)
ENDS	1.5	1.9	2.2	2.6	2.7	3.0	3.4	3.4	2.6	2.7	2.8	3.0	3.6	3.5	3.5	3.5	3.8	3.6	3.4	3.4	4.5 (2.1 to 7.0)^c^
Traditional SLT	10.3	10.0	9.7	10.0	10.1	9.8	9.5	9.9	10.9	10.0	10.1	10.4	10.3	9.9	9.6	9.9	10.1	9.7	9.6	9.9	−0.7 (−1.6 to 0.1)
Modern oral nicotine	0.1	0.1	0.1	0.1	0.2	0.3	0.5	0.7	0.9	0.9	1.1	1.3	1.6	1.7	2.1	2.4	2.6	2.8	3.1	3.4	23.2 (21.3 to 25.1)
Mean price adjusted for inflation, $																					
Cigarettes	5.73	5.88	5.91	5.95	6.03	6.13	6.26	6.34	6.35	6.58	6.72	6.76	7.01	7.38	7.50	7.72	8.08	8.41	8.51	8.55	2.1 (1.8 to 2.5)^c^
Cigars	1.19	1.23	1.23	1.21	1.23	1.23	1.26	1.25	1.28	1.34	1.38	1.39	1.43	1.52	1.52	1.53	1.60	1.68	1.68	1.66	1.9 (1.6 to 2.2)^c^
ENDS	10.81	11.61	12.23	12.59	12.82	12.51	11.75	11.14	11.38	11.67	11.45	10.97	11.26	11.99	12.51	13.18	14.29	15.01	15.87	16.42	2.2 (1.3 to 3.1)^c^
Traditional SLT	4.29	4.35	4.44	4.47	4.65	4.72	4.82	4.87	5.02	5.27	5.30	5.39	5.58	5.88	5.99	6.20	6.53	6.80	6.89	6.96	2.6 (2.2 to 3.0)^c^
MON	4.22	4.29	4.37	4.38	4.41	4.30	4.24	4.17	4.28	4.31	4.16	4.21	4.06	4.21	3.85	3.94	4.20	4.41	4.36	4.57	0.2 (−0.3 to 0.7)

Abbreviations: AQPC, average quarterly percent change; ENDS, electronic nicotine delivery systems; MON, modern oral nicotine; Q, quarter; SLT, smokeless tobacco.

^a^ Data are from the Nielsen Company through its Convenience Track Service for tobacco products.

^b^ Reported in billions; unit reflects how product is sold to consumer.

^c^ The AQPC is significantly different for 0 at *P* < .05.

**Figure.**
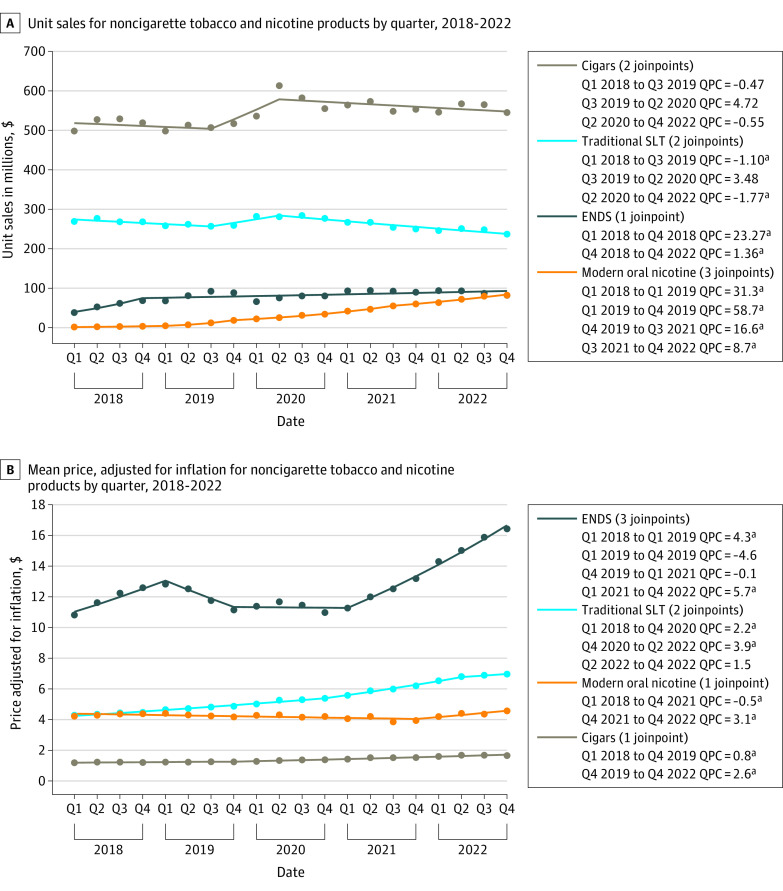
Quarterly Unit Sales and Mean Price for Noncigarette Tobacco and Nicotine Product by Type, 2018-2022 Unit reflects how product is sold to consumer (eg, pack of 20 cigarettes). ENDS indicates electronic nicotine delivery systems; Q, quarter; QPC, quarterly percent change; SLT, smokeless tobacco. ^a^*P* < .05.

Although findings are not reported by unit size, most sales by product are single packs for cigarettes (98.5%), single cans for SLT (94.2%) and MON (98.8%), and packs of 3 or fewer for cigars (86.4%). Greater variability was noted for ENDS because of the diverse subcategories (eg, disposable vs rechargeable). All products except MON significantly increased in price from 2018 to 2022 (Table). The most expensive products per unit were ENDS; cigars were the least expensive. ENDS prices significantly increased from 2018 to 2019, significantly decreased throughout 2019, and increased rapidly from 2021 to 2022. Unit prices for MON significantly decreased from 2018 to 2021 and then significantly increased (Figure, B).

## Discussion

Although cigarettes remained the dominant product sold during the study period, their market share decreased, leading to a more diverse marketplace at a range of price points. The fastest growing and only price-stable products were MON. The most expensive products were ENDS, yet prices decreased in 2019, coinciding with the emergence of cheap, flavored disposable vapes.^3^ The subsequent price increase may reflect a shrinking ENDS market given US Food and Drug Administration enforcement against illegal disposables. Cigars, which are largely flavored,^4^ remained the cheapest product, driven by low cost and small pack sizes, which is concerning given that flavored products and low prices are appealing to young people. A limitation of this study is that Nielsen data are obtained from convenience stores and may not be generalizable to all store types. However, most tobacco product sales occur in convenience stores.^5^

Faced with many products, consumers may be influenced by price differences to purchase products with varying levels of harm. In particular, the cheapness of cigars, which are combustible tobacco products with many of the same health risks as cigarettes,^6^ may perpetuate use. Policies that impact price (ie, taxation and minimum pack size) can discourage use of the most dangerous products and should be considered.

## Notes

Supplement.
Data Sharing Statement
