# Supplementary material for: National Trends in Sales and Price for Commercial Tobacco and Nicotine Products, 2018-2022
Source: JAMA Netw Open. 2024 Mar 7;7(3):e241384. doi: 10.1001/jamanetworkopen.2024.1384 (PMC10921248; doi:10.1001/jamanetworkopen.2024.1384)
Supplement: Supplement. — Data Sharing Statement [file jamanetwopen-e241384-s001.pdf]

Data Sharing Statement

Ganz. National Trends in Sales and Price for Commercial Tobacco and Nicotine Products, 2018-2022. *JAMA Netw Open*. Published March 07, 2024. doi:10.1001/jamanetworkopen.2024.1384

Data
Data available: No

Additional Information
Explanation for why data not available: Data from this study are available from Nielsen.
